# Correction: Integrated multi‑omics highlights alterations of gut microbiome functions in prodromal and idiopathic Parkinson’s disease

**DOI:** 10.1186/s40168-025-02295-4

**Published:** 2025-11-07

**Authors:** Rémy Villette, Júlia Ortís Sunyer, Polina V. Novikova, Velma T. E. Aho, Viacheslav A. Petrov, Oskar Hickl, Susheel Bhanu Busi, Charlotte De Rudder, Benoit J. Kunath, Anna Heintz‑Buschart, Jean‑Pierre Trezzi, Rashi Halder, Christian Jäger, Laura A. Lebrun, Annegrät Daujeumont, Sebastian Schade, Annette Janzen, Nico Jehmlich, Martin von Bergen, Cédric C. Laczny, Patrick May, Claudia Trenkwalder, Wolfgang Oertel, Brit Mollenhauer, Paul Wilmes

**Affiliations:** 1 Luxembourg Centre for Systems Biomedicine, University of Luxembourg, Esch‑Sur‑Alzette, Luxembourg; 2 UK Centre for Ecology and Hydrology, Wallingford, Oxfordshire UK; 3 Biosystems Data Analysis, Swammerdam Institute for Life Sciences, University of Amsterdam, Amsterdam, The Netherlands; 4 Department of Neurology, University Medical Center Göttingen, Göttingen, Germany; 5 Paracelsus-Elena-Klinik, Kassel, Germany; 6 Department of Neurology, Philipps-University Marburg, Marburg, Germany; 7 Department of Molecular Toxicology, Helmholtz-Centre for Environmental Research GmbH – UFZ, Leipzig, Germany; 8 Department of Neurosurgery, University Medical Center Göttingen, Göttingen, Germany; 9 Department of Life Sciences and Medicine, Faculty of Science, Technology and Medicine, University of Luxembourg, Belvaux, Luxembourg

**Correction** **:** **Microbiome 13, 200 (2025)**

**https://doi.org/10.1186/s40168-025-02227-2**

Following publication of the original article [1], the authors reported that the Data Availability section was incorrectly revised following the proofing stage by the Production team. The correct section is as follows:

Metagenomic and metatranscriptomic data are available on SRA, under the BioProject: PRJNA782492. The mass spectrometry proteomics data have been deposited to the ProteomeXchange Consortium via the PRIDE partner repository with the dataset identifier PXD031457. The IMP pipeline, which was used for analysis of metagenomic and metatranscriptomic data, is available at https://gitlab.lcsb.uni.lu/IMP/imp3. The R and python code used for statistical analyses and visualizations is available at https://gitlab.com/uniluxembourg/lcsb/systems-ecology/multi_omics_of_pd_and_irbd. Metadata are available via the ELIXIR Luxembourg Data Catalogue: https://datacatalog.elixir-luxembourg.org/e/dataset/ELU-2-81D473-1.

The original article has been updated.
