# Supplementary figures and images for: Integrated multi-omics highlights alterations of gut microbiome functions in prodromal and idiopathic Parkinson’s disease
Source: Microbiome. 2025 Oct 7;13:200. doi: 10.1186/s40168-025-02227-2 (PMC12502400; doi:10.1186/s40168-025-02227-2)

**A**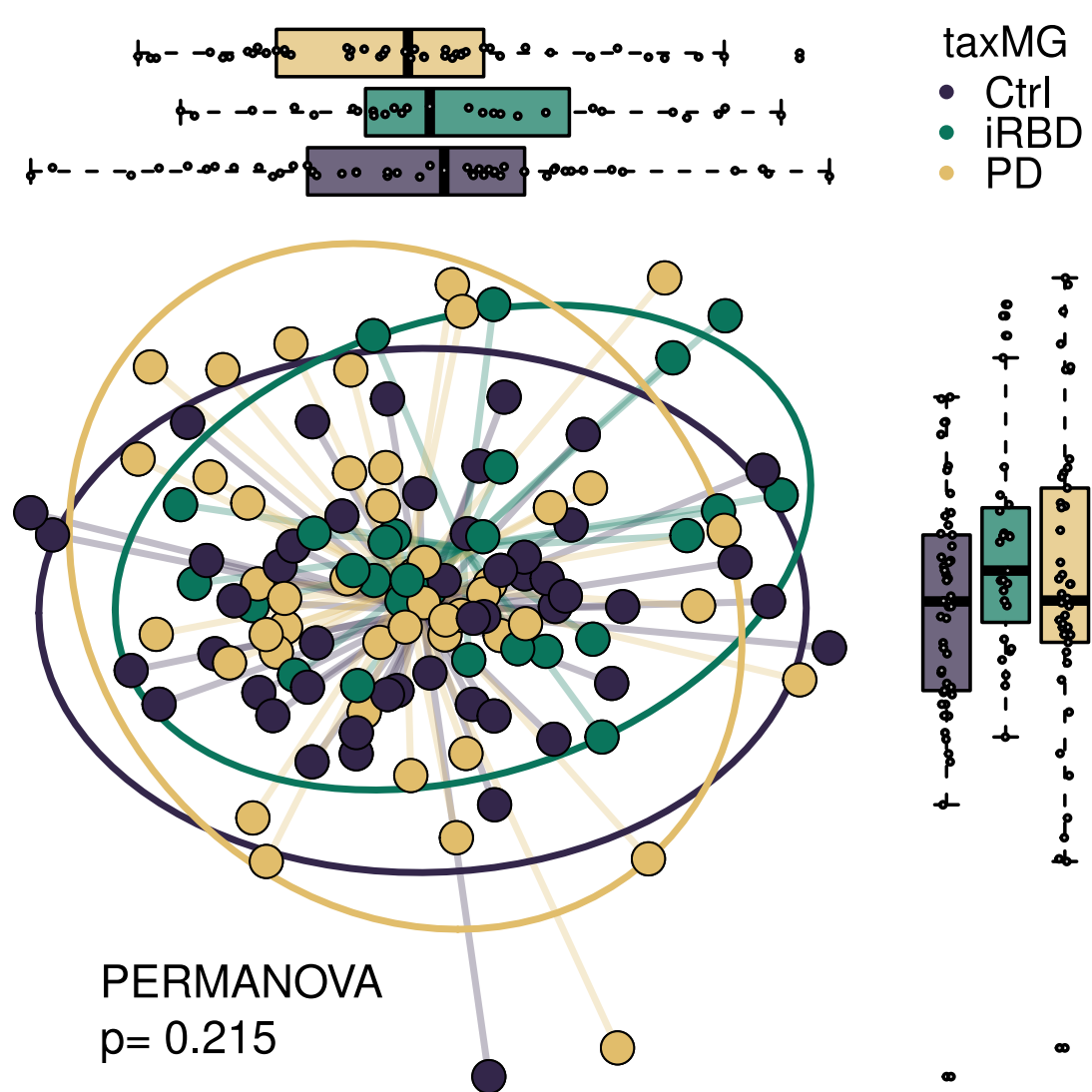**B**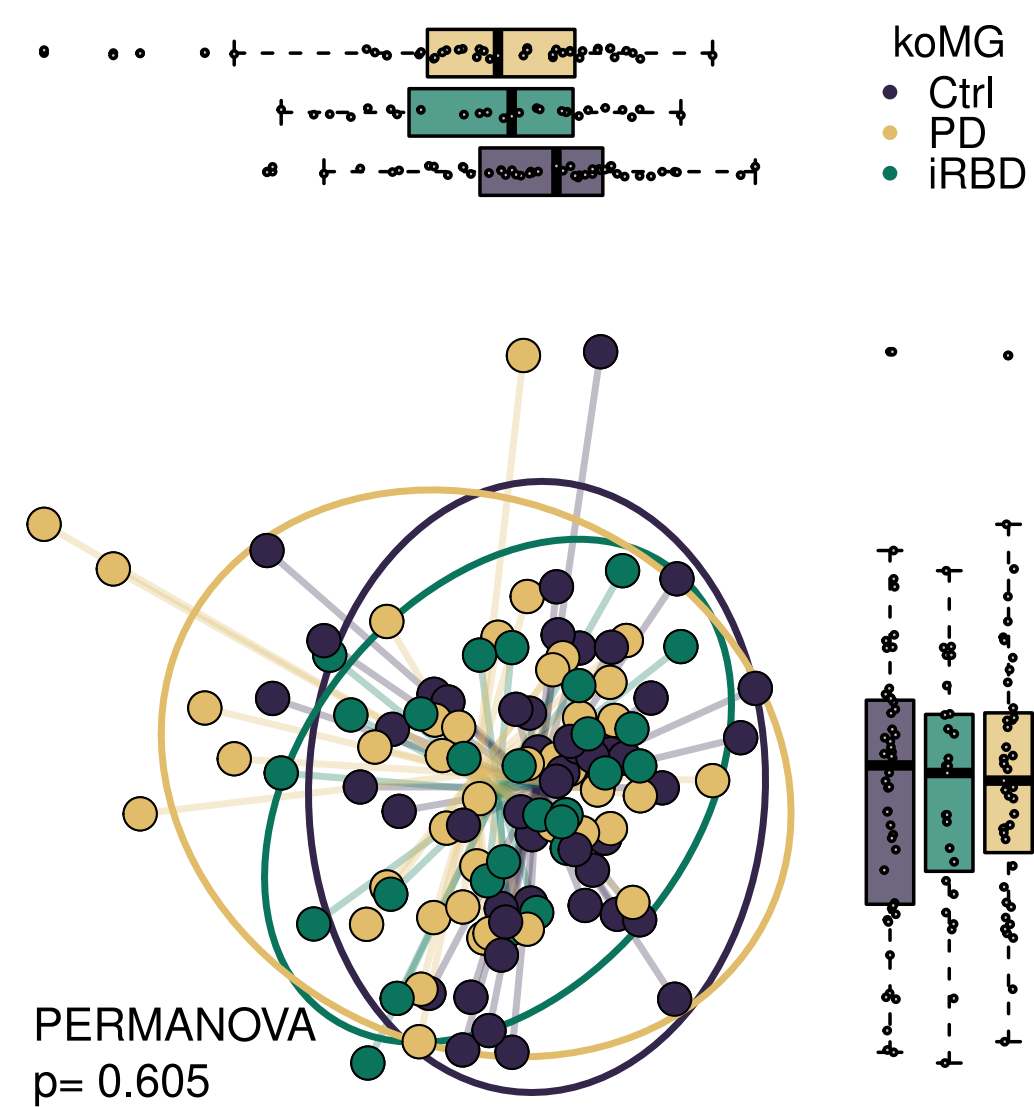**C**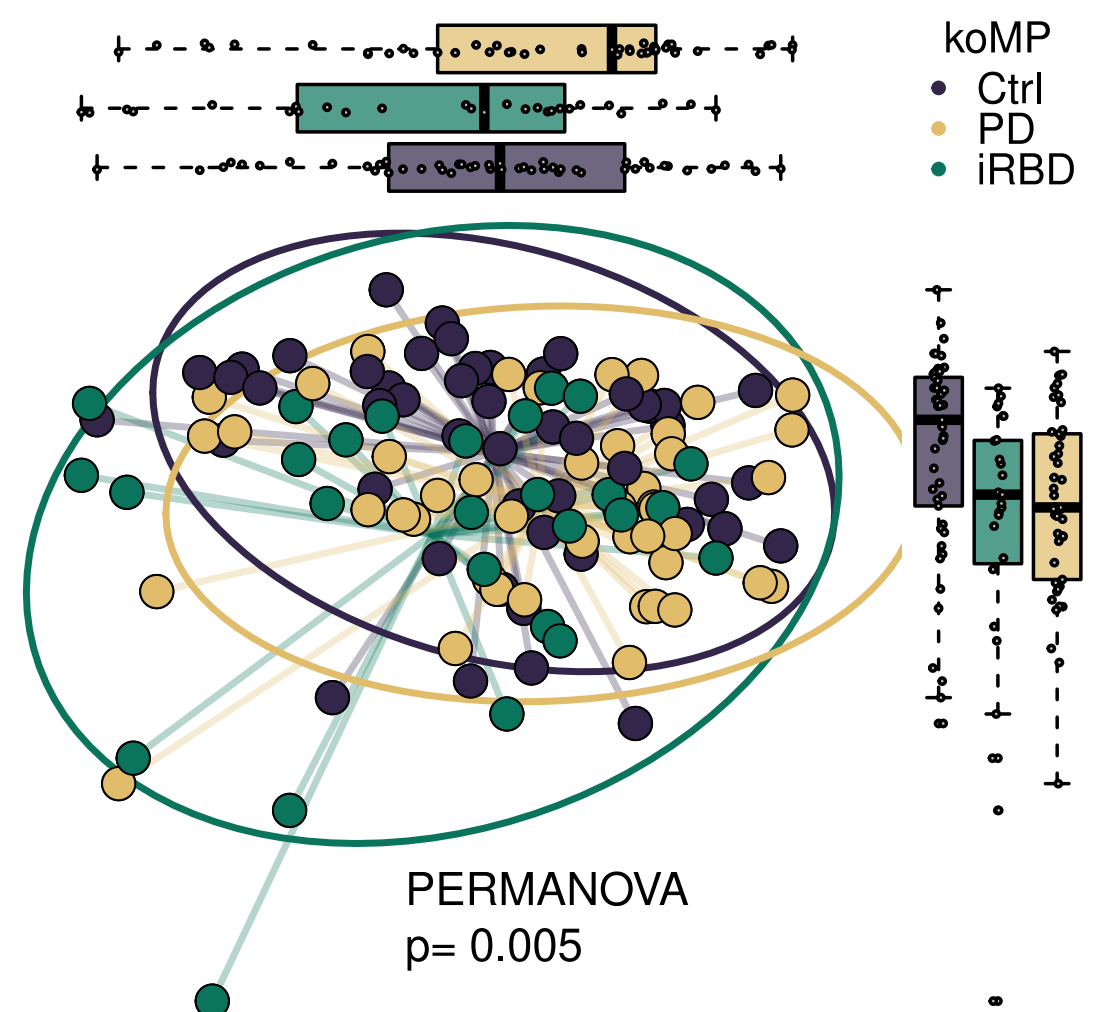**D**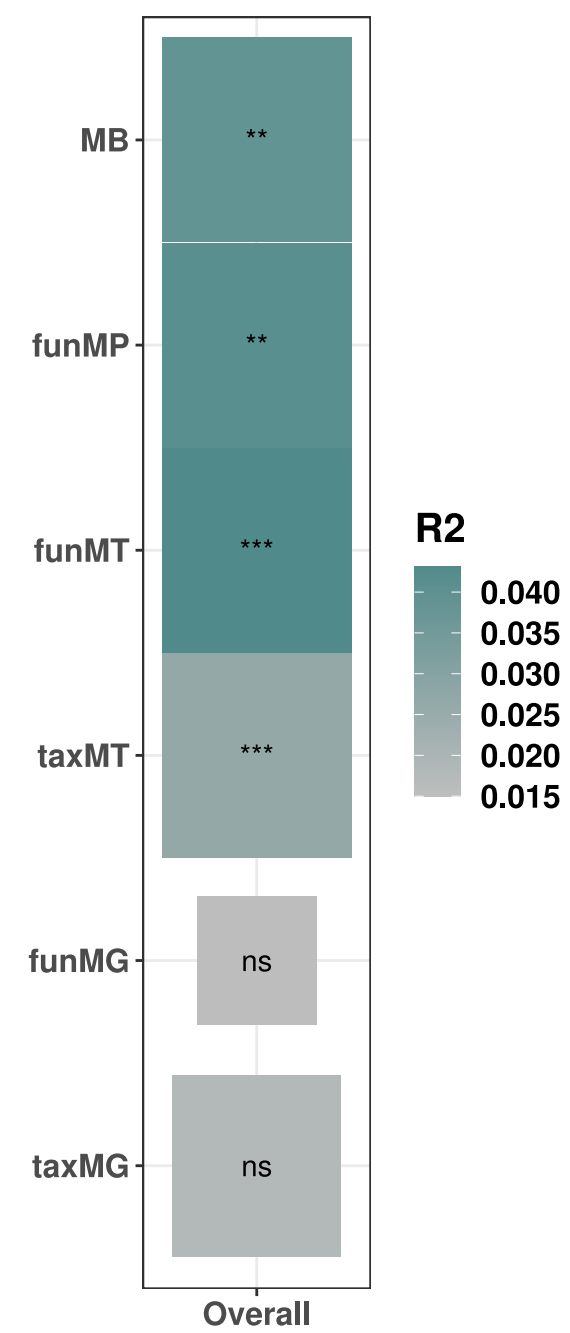

Supplement: Supplementary file 1 — Supplementary Material 1: Extended figure 1. NMDS analysis of A. metagenomic taxonomic composition (taxMG), B. metagenomic functions (funMG) and C. meta-proteomic taxonomic composition (taxMP), using a Bray-Curtis dissimilarity matrix. D. PCA analysis of metaproteomic functions (funMP). E. PERMANOVA analysis for the three groups and all omics. Colour represents R² values and size is–log10(p-value). All PERMANOVA analysis were run using 1000 permutations using a Bray-Curtis dissimilarity matrix. [file 40168_2025_2227_MOESM1_ESM.pdf]

A

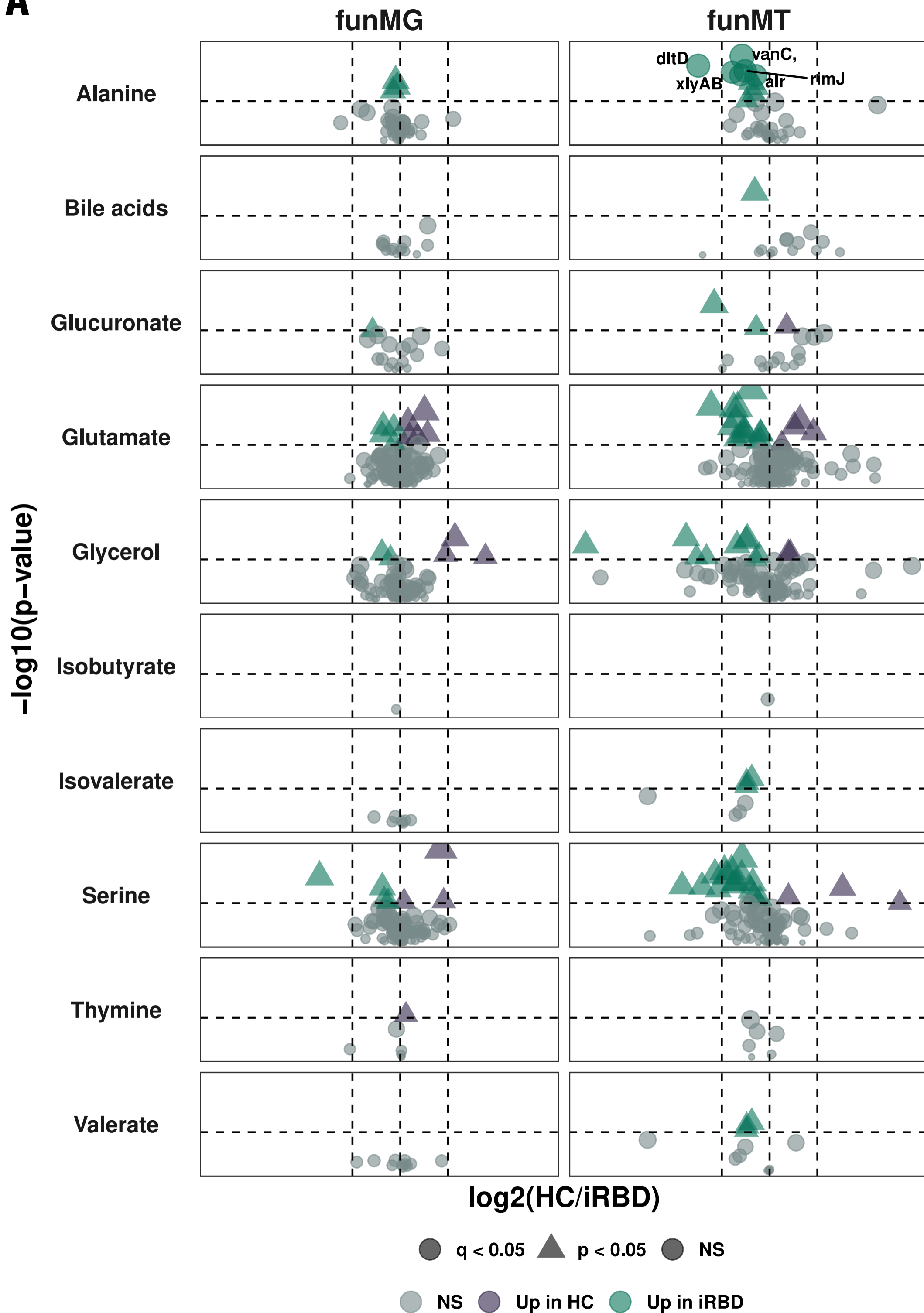

B

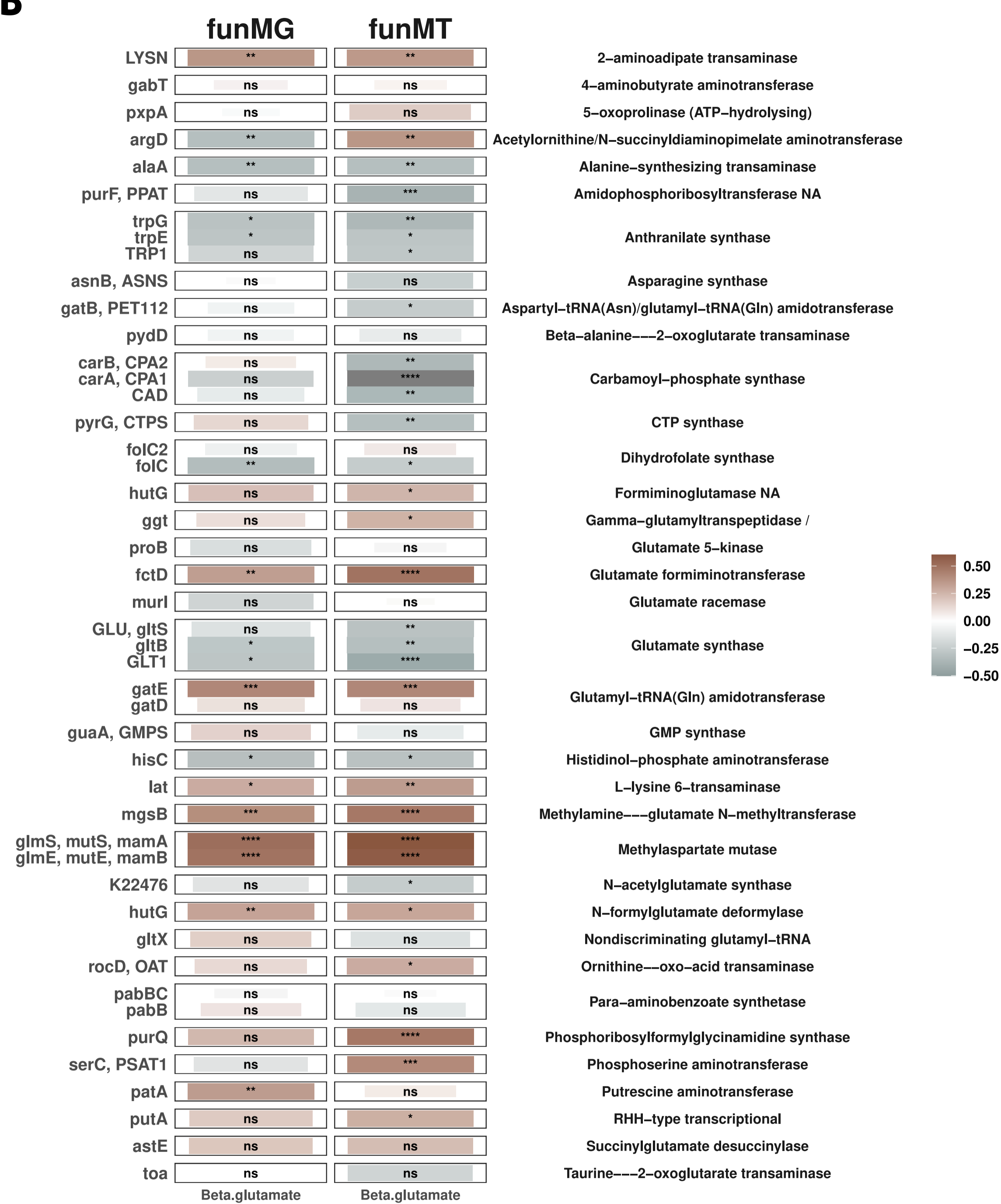

Supplement: Supplementary file 4 — Supplementary Material 4: Extended figure 4. A. Absolute log2 fold change between HC and iRBD for funMG and funMT associated to significant compounds. Dots are scaled by the –log10(p-value), colorized and shaped according to p-value significance before (triangle shape) and after FDR correction (round shape). B. Spearman correlation between beta-glutamate relative abundance and funMG-funMT KEGG orthologs related to glutamate species. Only genes with at least one significant correlation are plotted. All p-values are FDR corrected. [file 40168_2025_2227_MOESM4_ESM.pdf]

A

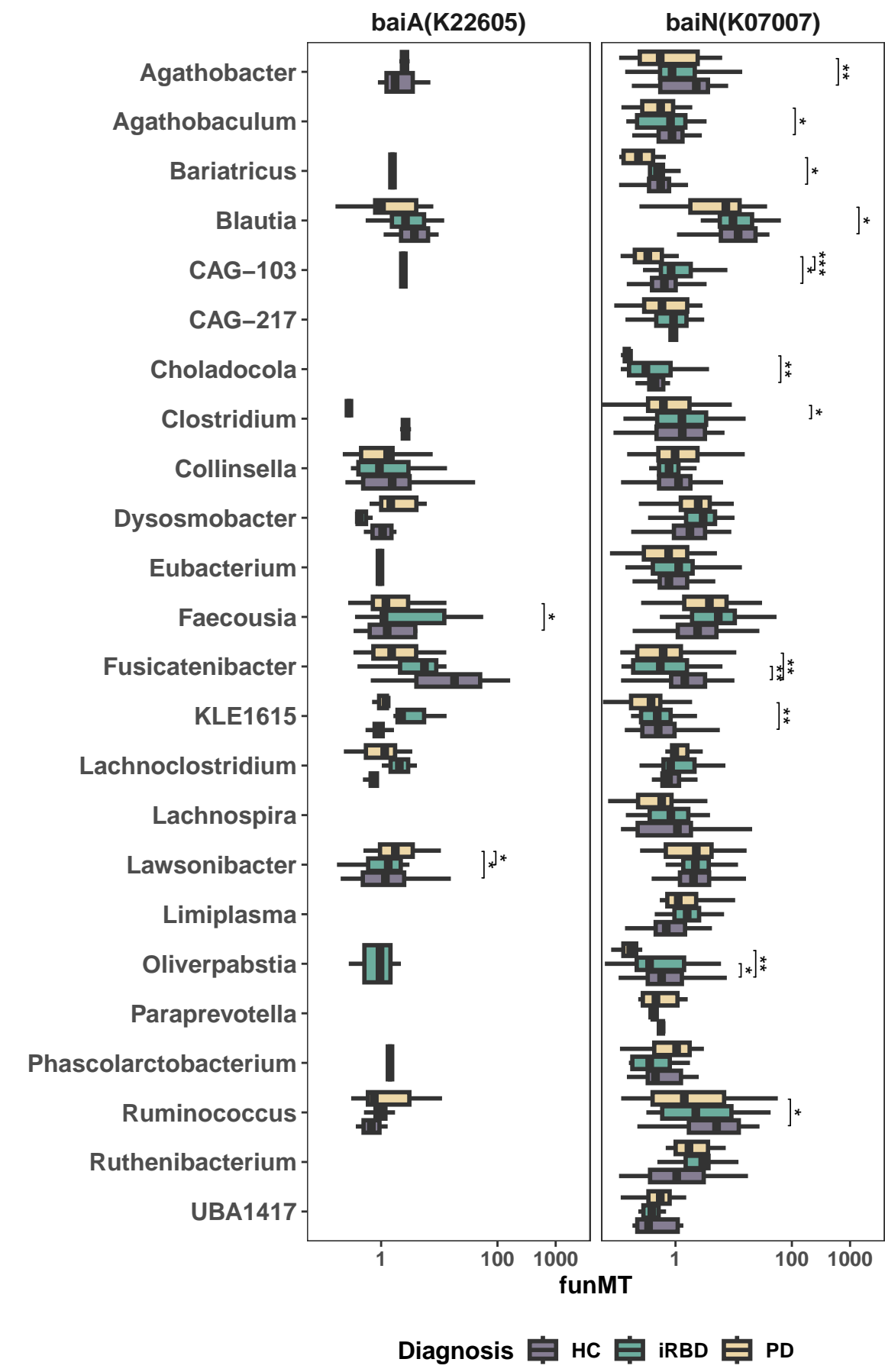

B

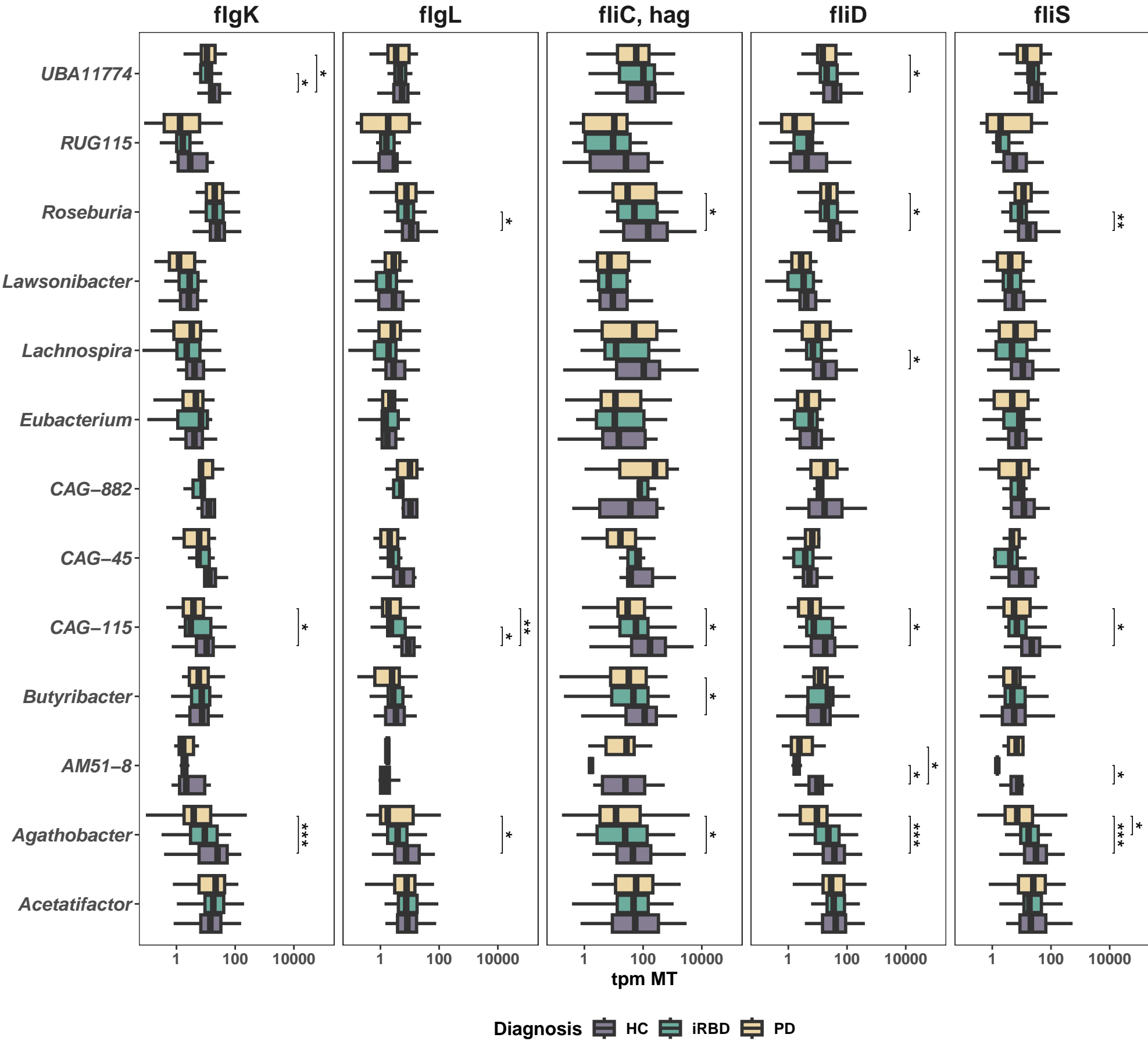

Supplement: Supplementary file 5 — Supplementary Material 5: Extended figure 5. A. Bile acids transcripts found significantly different between the groups. P-values are corrected with FDR. B. Flagellar assembly transcripts encoding for extracellular component of the flagella for the genus present in Cluster 2. All tests are Wilcoxon tests. [file 40168_2025_2227_MOESM5_ESM.pdf]

Multiomics variance explained by MoFa factors

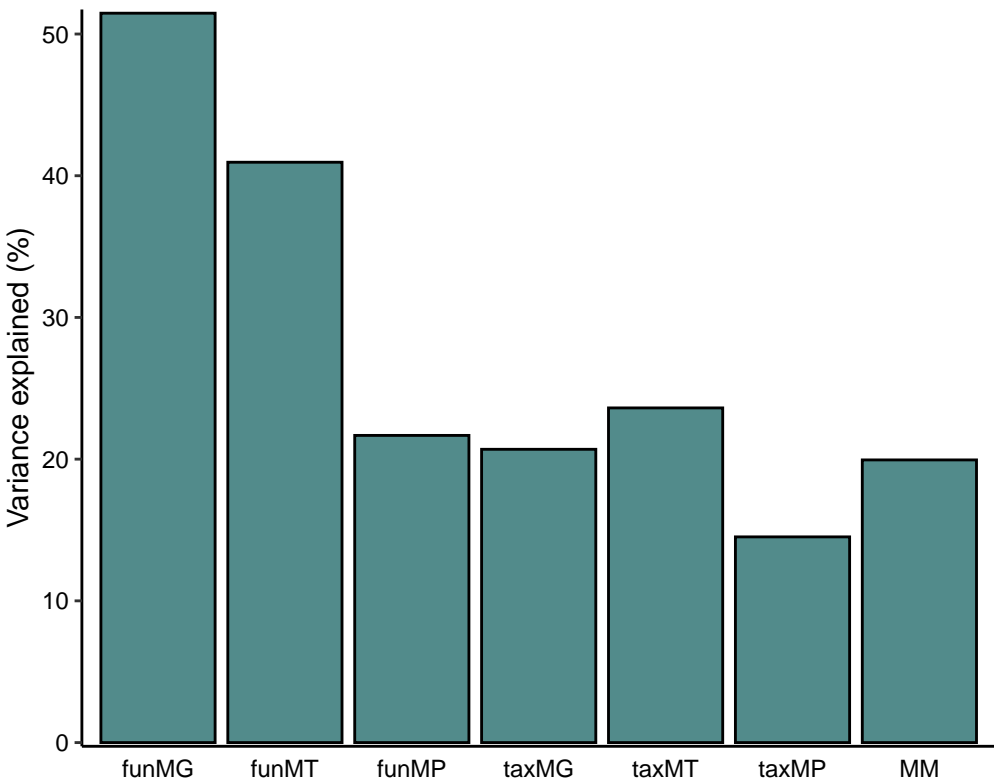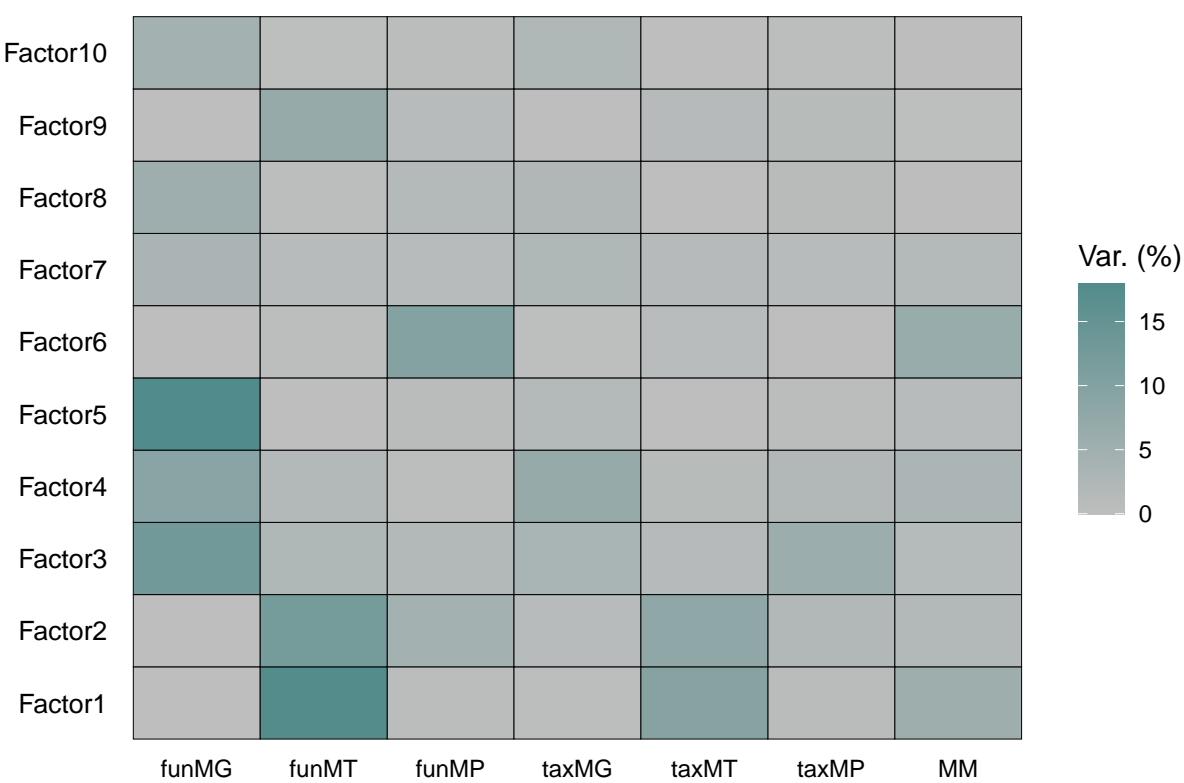

Supplement: Supplementary file 6 — Supplementary Material 6: Extended figure 6. Multiomics variance explained by MOFA factors. A. Variance explained by the MOFA factors across different omics layers, total. B. Variance explained by the MOFA factors across different omics layers, split by factors. [file 40168_2025_2227_MOESM6_ESM.pdf]
