# Supplementary material for: Integrated multi-omics highlights alterations of gut microbiome functions in prodromal and idiopathic Parkinson’s disease
Source: Microbiome. 2025 Oct 7;13:200. doi: 10.1186/s40168-025-02227-2 (PMC12502400; doi:10.1186/s40168-025-02227-2)
Supplement: Supplementary file 2 — Supplementary Material 2: Extended figure 2. A. Differential abundance analysis at the genus level using SIAMCAT algorithm. B. Differential abundance analysis using ALDEx2 algorithm at the species level. Values are pseudo fold changes for HC/PD and size is based on –log10(p-value). Shape is referring to level of significance, triangular shape for p-value significance before and round shape for p-values < 0.05 after FDR correction. C to F ALDEx2 differential abundance analysis on funMG for HC vs PD (C) and HC vs iRBD (D); funMT for HC vs PD (E) and HC vs iRBD (F). All genes and transcripts are colored and shaped according to p-value significance before (triangle shape) and after FDR correction (round shape). [file 40168_2025_2227_MOESM2_ESM.pdf]

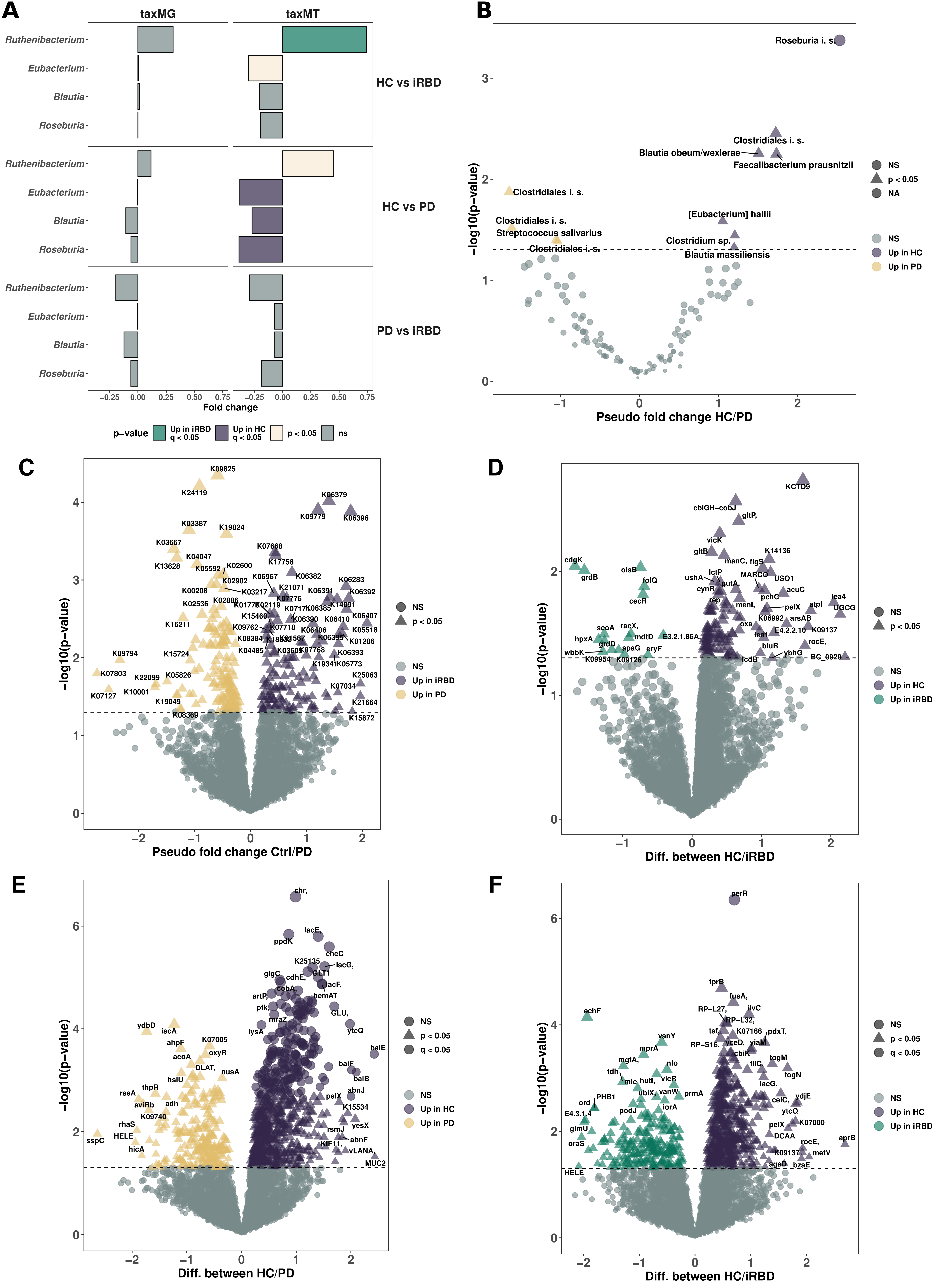

A
taxMG
taxMT
Ruthenibacterium
Eubacterium
Blautia
Roseburia
HC vs iRBD
HC vs PD
PD vs iRBD
Fold change
p-value
Up in iRBD q < 0.05
Up in HC q < 0.05
p < 0.05
ns
B
Roseburia i. s.
Clostridiales i. s.
Blautia obeum/wexlerae
Faecalibacterium prausnitzii
Clostridiales i. s.
[Eubacterium] hallii
Clostridiales i. s.
Streptococcus salivarius
Clostridium sp.
Clostridiales i. s.
Blautia massiliensis
-log10(p-value)
Pseudo fold change HC/PD
NS
p < 0.05
NA
NS
Up in HC
Up in PD
C
Pseudo fold change Ctrl/PD
NS
p < 0.05
NS
Up in iRBD
Up in PD
D
Diff. between HC/iRBD
NS
p < 0.05
NS
Up in HC
Up in iRBD
E
Diff. between HC/PD
NS
p < 0.05
q < 0.05
NS
Up in HC
Up in PD
F
Diff. between HC/iRBD
NS
p < 0.05
q < 0.05
NS
Up in HC
Up in iRBD
