# Supplementary material for: Integrated multi-omics highlights alterations of gut microbiome functions in prodromal and idiopathic Parkinson’s disease
Source: Microbiome. 2025 Oct 7;13:200. doi: 10.1186/s40168-025-02227-2 (PMC12502400; doi:10.1186/s40168-025-02227-2)
Supplement: Supplementary file 3 — Supplementary Material 3: Extended figure 3. A. Percentage of variance explained for each metabolite for “Sex” and “Diagnosis” and B. Percentage of variance explained for each metabolite for “Constipation” and “Diagnosis” (right panel). Metabolites include untargeted meta-metabolomics, targeted SCFA and targeted bile acids, normalized by sum before merging and variance quantification. C. Variance for each metabolite associated to the clinical factors “Diagnosis”, “Sex” and “Constipation”. Metabolites include untargeted meta-metabolomics, targeted SCFA and targeted bile acids, normalized by sum before merging and variance quantification. D. Spearman correlation between metabolites and taxMG species. P-values are FDR corrected. [file 40168_2025_2227_MOESM3_ESM.pdf]

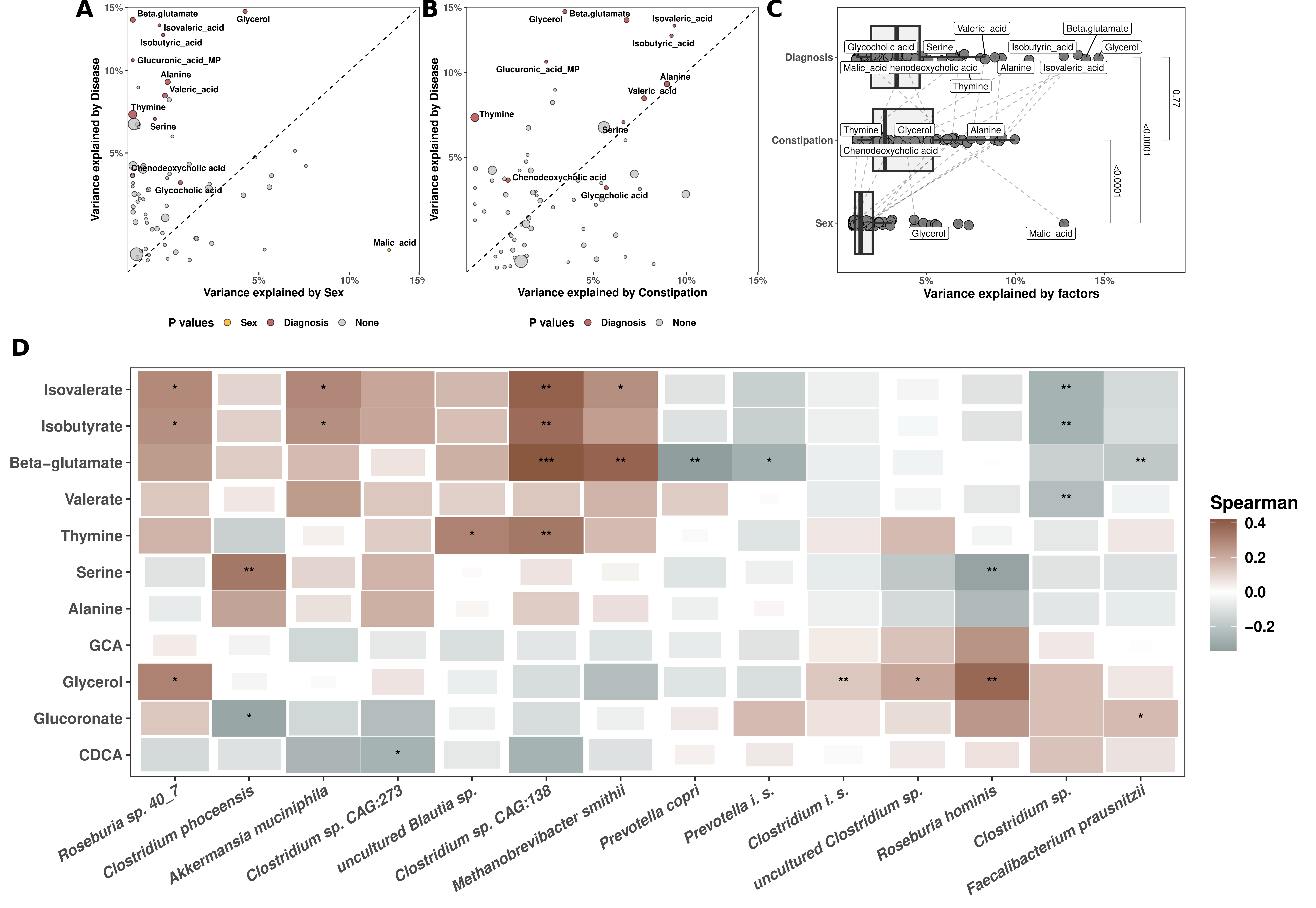

A
B
C
D
Variance explained by Disease
Variance explained by Sex
Variance explained by Constipation
Variance explained by factors
15%
10%
5%
Beta.glutamate
Glycerol
Isovaleric_acid
Isobutyric_acid
Glucuronic_acid_MP
Alanine
Valeric_acid
Thymine
Serine
Chenodeoxycholic acid
Glycocholic acid
Malic_acid
Serine
Diagnosis
Constipation
Sex
0.77
<0.0001
<0.0001
P values
Sex
Diagnosis
None
Spearman
0.4
0.2
0.0
−0.2
Isovalerate
Isobutyrate
Beta−glutamate
Valerate
Thymine
Serine
Alanine
GCA
Glycerol
Glucoronate
CDCA
Roseburia sp. 40_7
Clostridium phoceensis
Akkermansia muciniphila
Clostridium sp. CAG:273
uncultured Blautia sp.
Clostridium sp. CAG:138
Methanobrevibacter smithii
Prevotella copri
Prevotella i. s.
Clostridium i. s.
uncultured Clostridium sp.
Roseburia hominis
Clostridium sp.
Faecalibacterium prausnitzii
